# *ACTA1*-related congenital myopathy in a neonate: a case report and literature review

**DOI:** 10.3389/fped.2025.1706982

**Published:** 2026-01-20

**Authors:** Lingxia Zhao, Feng Deng, Baohuan Cai

**Affiliations:** 1 Department of Neonatology, Wuhan Children’s Hospital (Wuhan Maternal and Child Healthcare Hospital), Tongji Medical College, Huazhong University of Science and Technology, Wuhan, China; 2 Department of Pediatrics, Qianjiang Maternal and Child Health Family Planning Service Center, Qianjiang, China; 3 Department of Pediatrics, Tongji Hospital, Tongji Medical College, Huazhong University of Science and Technology, Wuhan, China

**Keywords:** *ACTA1* gene, congenital myopathy, hypoxic-ischemic encephalopathy, neonatal hypotonia, skeletal muscle α-actin

## Abstract

**Background:**

*ACTA1*-related congenital myopathies are rare neuromuscular disorders with significant genotypic heterogeneity, often causing severe neonatal multisystem involvement. This study presents a severe neonatal case with a pathogenic *ACTA1* variant and reviews literature to highlight diagnostic and management challenges.

**Case presentation:**

A female infant was born via cesarean section at 39^+1^ weeks to a non-consanguineous mother. Prenatal ultrasound showed polyhydramnios. She presented with severe birth asphyxia (Apgar 3 at 1 min, 5 at 5 min), requiring immediate resuscitation. Physical examination revealed profound hypotonia, absent spontaneous movements, respiratory insufficiency necessitating mechanical ventilation, expressionless facies, and bulbar dysfunction. Laboratory tests indicated metabolic acidosis and elevated lactate and creatine kinase. Electromyography (EMG) demonstrated reduced motor amplitudes and spontaneous fibrillations. Whole-exome sequencing identified a *de novo* heterozygous pathogenic variant in *ACTA1* (c.227G>A, p. Gly76Asp), confirming *ACTA1*-related congenital myopathy. Care was withdrawn on day 18 due to poor neurologic recovery.

**Conclusion:**

This case highlights three critical implications: (1) the significant clinical overlap between *ACTA1* myopathy and perinatal asphyxia, underscoring the necessity of genetic testing in hypotonic neonates with atypical presentations; (2) the grave prognosis of early-onset *ACTA1* mutations, which mandates early palliative care consultation; and (3) the essential role of a precise genetic diagnosis in defining phenotypes and informing future targeted therapies, such as gene therapy.

## Introduction

Congenital myopathies represent a heterogeneous group of inherited neuromuscular disorders characterized by structural abnormalities in skeletal muscle fibers. They typically present with hypotonia, muscle weakness, and respiratory insufficiency beginning in infancy or early childhood (1). Among the various genetic causes, mutations in the *ACTA1* gene, which encodes skeletal muscle *α*-actin, account for approximately 15%–25% of cases. These mutations are associated with distinct histopathological subtypes, including nemaline myopathy, actin aggregate myopathy, and congenital fiber-type disproportion (CFTD) (2, 3).

Neonatal-onset *ACTA1*-related myopathy is particularly severe, often manifesting as fetal akinesia, arthrogryposis, polyhydramnios, and immediate respiratory failure at birth (4). Despite advances in genetic diagnostics, the condition is frequently misdiagnosed as hypoxic-ischemic encephalopathy (HIE) or spinal muscular atrophy due to overlapping clinical features (5). This diagnostic delay impedes timely genetic counseling and complicates prognostication and family planning. The prognosis is generally poor, with over 40% of neonatal cases succumbing to respiratory complications within the first year of life (6). Given the severity, phenotypic variability, and challenges in early recognition, there is a continued need to delineate the clinical and genetic spectrum of this disorder.

In this article, we report a severe neonatal case caused by a pathogenic *ACTA1* variant, and provide a comprehensive review of previously reported cases to improve awareness, facilitate early diagnosis, and highlight multidisciplinary management challenges.

## Case presentation

**Figure 1:**
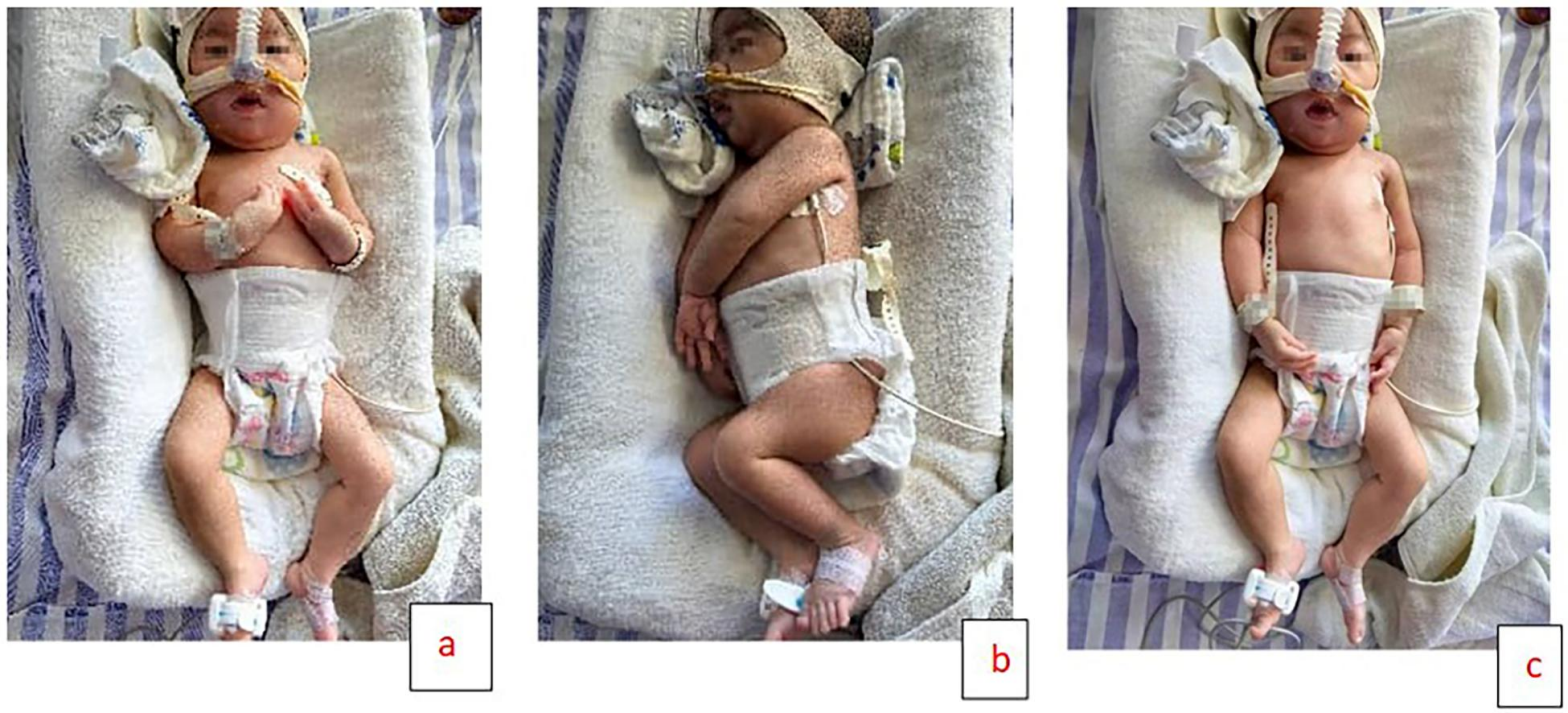
Three-panel image of a newborn undergoing medical treatment. Panel **a** and **c** show the baby lying on their back with monitoring devices attached. Panel **b** shows the baby lying on their side with similar equipment.

The proband was a female infant born at 39^+1^ weeks via cesarean section to a 29-year-old G3P2 mother with a history of uterine scarring. Prenatal ultrasound detected polyhydramnios (amniotic fluid volume: 2,500 ml), but no other structural anomalies were identified. There was no consanguinity between parents, and family history was negative for neuromuscular or metabolic disorders. The proband's 7-year-old sibling exhibited normal motor development. At delivery, the infant demonstrated severe birth asphyxia, with Apgar scores of 3 and 5 at 1 and 5 min, respectively, necessitating immediate resuscitation. Upon transfer to the neonatal intensive care unit, she exhibited lethargy, poor responsiveness, and an expressionless facies. Neuromuscular examination revealed profound generalized hypotonia and absence of spontaneous movements, with only minimal finger and toe motion observed. She developed respiratory insufficiency requiring mechanical ventilation. Bulbar dysfunction was prominent, manifesting as weak sucking and swallowing reflexes along with excessive oral secretions. Primitive reflexes including Moro and grasp were absent, although ocular movements remained intact (Figure 1).

**Figure 2:**
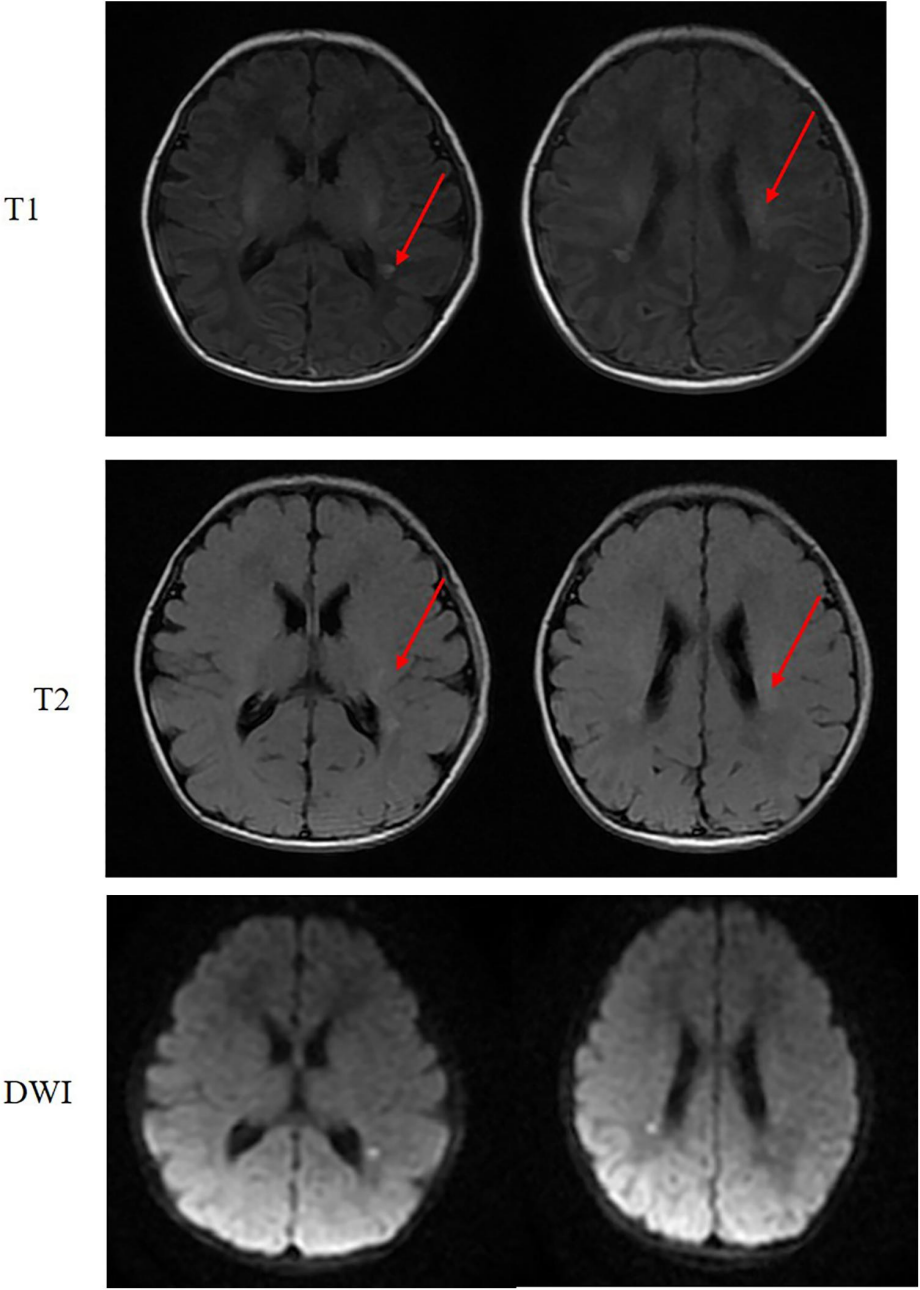
Axial MRI of the brain. The image demonstrates punctate lesions with short T1 and short T2 signal intensity (arrows) adjacent to the bilateral lateral ventricles. These lesions show restricted diffusion on diffusion weighted imaging (DWI) mapping.

Initial laboratory studies revealed metabolic acidosis (pH 7.27, pCO₂ 46.2 mmHg, HCO₃⁻ 20 mmol/L, SBE −5.3 mmol/L) with elevated lactate (2.7 mmol/L). Cardiac evaluation demonstrated markedly elevated creatine kinase (peak 578 U/L, normalizing by Day 9), while electrocardiography (ECG) showed sinus tachycardia (177 bpm) with ventricular ectopy. Echocardiographic findings were notable for a hemodynamically insignificant patent ductus arteriosus (1.2 mm) with preserved left ventricular function (ejection fraction 53%). Neurophysiological assessment identified multifocal epileptiform discharges and subclinical seizures on Day 1 electroencephalography (EEG), with subsequent electromyography (EMG) on Day 10 showing reduced motor amplitudes (median nerve: right 0.64 mV, left 1.94 mV) and spontaneous fibrillations in limb muscles. Brain magnetic resonance imaging (MRI) demonstrated periventricular T1/T2 hyperintensities with diffusion restriction, though no structural anomalies were observed (Figure 2).

**Figure 3:**
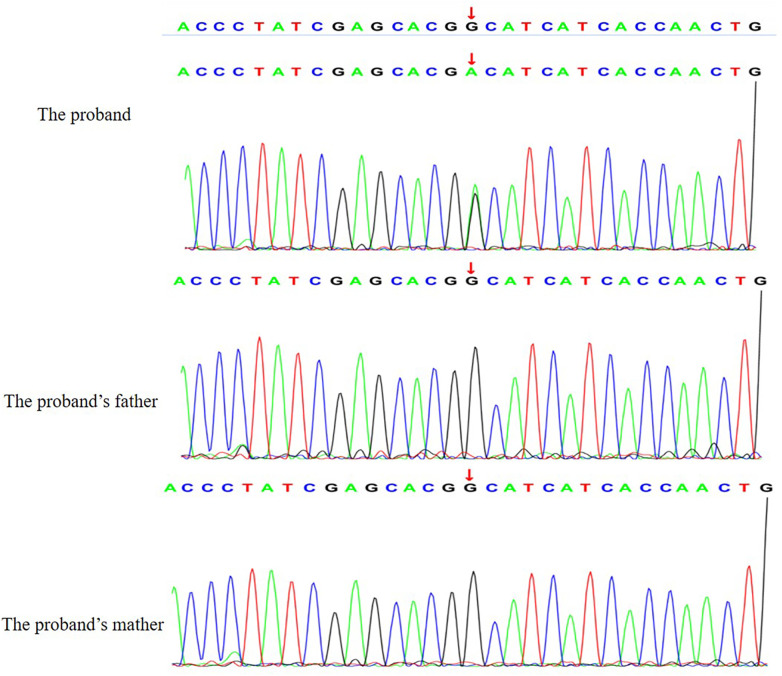
The validation results by sanger sequencing for the *ACTA1* variants.

Whole-exome sequencing (WES) was performed to investigate the underlying genetic etiology of the patient's severe neonatal presentation. WES identified a heterozygous missense variant in the *ACTA1* gene, specifically at position c.227G>A, resulting in the amino acid substitution

p. Gly76Asp. This variant was not detected in either parent via Sanger sequencing, confirming its *de novo* origin. In accordance with the American College of Medical Genetics and Genomics (ACMG)/Association for Molecular Pathology (AMP) guidelines, the variant was classified as pathogenic. The evidence supporting this classification included the following: PS2 (*de novo* occurrence in a patient with the disease and no family history); PM1 (located in a mutational hot spot and/or critical functional domain well-established in disease pathology); PM2 (absent or very low frequency in population databases such as gnomAD); and PP3 (multiple computational predictions support a deleterious effect on the gene or gene product). Seen in Figure 3.

**Figure 4:**
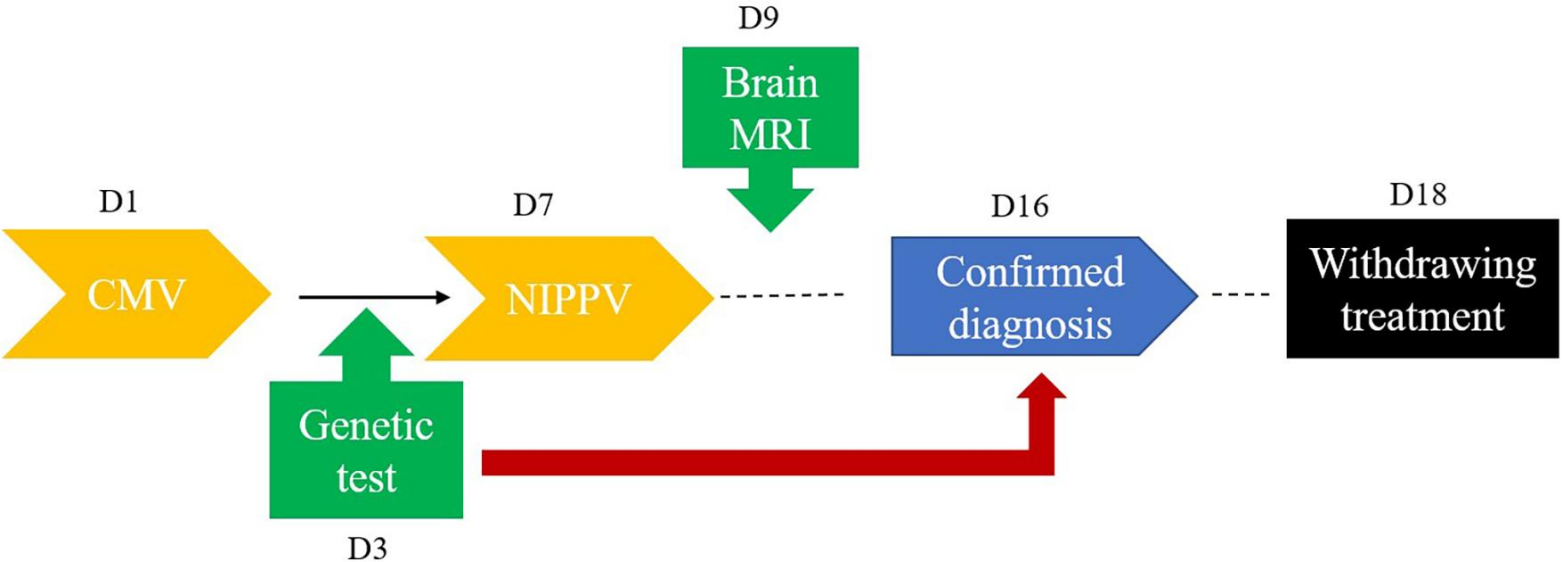
The timeline for clinical events. CMV: conventional mechanical ventilation.

The infant demonstrated a complicated clinical course, requiring prolonged respiratory support—initially with 7 days of mechanical ventilation, followed by unsuccessful attempts at weaning to non-invasive positive pressure ventilation (NIPPV). Neuroprotective measures, including therapeutic hypothermia and vitamin B1/B12 supplementation, were implemented to mitigate neurological injury. Swallowing rehabilitation therapy was initiated but yielded minimal functional improvement. Although serial EEG monitoring showed gradual improvement by Day 7, the infant exhibited persistent, profound neuromuscular weakness inconsistent with typical hypoxic-ischemic encephalopathy recovery. Given the poor prognosis and lack of meaningful neurological recovery, the family elected to withdraw life-sustaining care on Day 18, and the infant passed shortly thereafter. A schematic overview of the clinical events development is provided in Figure 4.

## Discussion

This case illustrates a severe neonatal presentation of *ACTA1*-related myopathy, associated with a *de novo* heterozygous pathogenic variant (c.227G>A, p. Gly76Asp) in the *ACTA1* gene. The infant exhibited classic features including profound hypotonia, respiratory failure, bulbar dysfunction, arthrogryposis, and an expressionless facies, consistent with previously reported cases of neonatal-onset actinopathy (7–11). Notably, the initial clinical picture complicated the diagnostic process due to significant overlap with hypoxic-ischemic encephalopathy (HIE), particularly given the observed perinatal asphyxia and metabolic acidosis. However, the persistence of severe neuromuscular weakness beyond the typical recovery window for HIE, along with the absence of significant hypoxic injury on neuroimaging, raised suspicion of an underlying neuromuscular disorder. This diagnostic challenge underscores the necessity of including genetic myopathies in the differential diagnosis of floppy infants, even with suggestive perinatal complications.

Genetic testing played a pivotal role in confirming the diagnosis. WES identified the missense variant p. Gly76Asp, affecting a highly conserved residue within the skeletal muscle α-actin protein. Previous functional studies of variants at this residue and adjacent amino acids have demonstrated detrimental effects on actin polymerization and filament stability, further corroborating its pathogenicity (12). The genetic inheritance of *ACTA1*-related disorders is complex, predominantly autosomal dominant. In this pattern, a pathogenic variant in a single allele (heterozygosity) is sufficient to result in the disease phenotype. This genetic finding is consistent with the severe clinical phenotype observed in the proband. In accordance with ACMG guidelines, the variant was classified as pathogenic based on several criteria: its *de novo* occurrence (PS2), low population frequency (PM2), localization within a critical functional domain (PM1), and supportive computational evidence of deleterious impact (PP3) (7, 13–17). The absence of the variant in both parents—confirmed via Sanger sequencing—further supports its *de novo* origin, which is a common inheritance pattern in severe neonatal cases (16, 17). The predominance of missense mutations, distributed throughout the gene without a clear hotspot, is well-documented in *ACTA1*-related disease, and this case reinforces that observation (7, 13–15).

The multisystemic manifestations observed here highlight the severe functional impact of *ACTA1* mutations on striated muscle and related systems. Beyond generalized weakness and respiratory failure, the patient also showed elevated creatine kinase levels and cardiac ectopy, suggesting subclinical cardiac involvement—a finding occasionally noted in severe phenotypes (7, 9). Additionally, the combination of hypertonic contractures against a background of global hypotonia illustrates the complex and often paradoxical effects of actin dysfunction on muscle tone and contractility. Tragically, the clinical course in this case was fatal, consistent with the poor prognosis associated with neonatal-onset *ACTA1* myopathies. As reported in the literature, such patients often experience rapid progression of respiratory insufficiency leading to early mortality, despite maximal respiratory and nutritional support (7, 11, 18). This outcome reinforces the devastating natural history of this condition and highlights the limited efficacy of current supportive interventions in altering disease progression in the most severe forms. The family's decision to withdraw life-sustaining care, though profoundly difficult, is not uncommon in such contexts and reflects the severe neurodevelopmental prognosis and lack of disease-modifying therapies (10, 11, 19).

This study has several limitations that warrant discussion. First, as a single-center case report, our conclusions are based on the observation of a solitary patient. Given the significant phenotypic heterogeneity associated with *ACTA1*-related myopathy, the generalizability of these findings to the broader patient population should be interpreted with caution. Second, this study lacks independent verification via muscle biopsy, which remains the traditional diagnostic gold standard. However, in the contemporary era of advanced molecular diagnostics, genetic testing has become a central pillar for the definitive diagnosis and classification of congenital myopathies, providing a reliable diagnostic pathway for critically ill patients where invasive biopsy is not feasible. Despite these limitations, the detailed clinical course and genetic information documented in this case provide a crucial natural history dataset and phenotypic framework. Current management of *ACTA1*-related myopathy remains primarily supportive; however, a deeper understanding of its pathological mechanisms—specifically delineating between loss-of-function and toxic gain-of-function effects—is driving a paradigm shift toward etiology-targeted treatments (1, 3). For loss-of-function mutations, adeno-associated virus (AAV)-mediated gene replacement therapy represents a highly promising strategy (20). The data from this report are essential for designing future clinical trials of such advanced therapies, particularly for application in the most severe neonatal population, as they can inform patient stratification, biomarker selection, and endpoint determination.

## Conclusion

This case highlights the critical role of early genetic testing, such as rapid WES, in the diagnostic evaluation of neonates with unexplained hypotonia and respiratory failure. A definitive genetic diagnosis is essential to guide clinical management, facilitate accurate prognostic counseling, and inform family-centered decision-making. Future efforts should focus on elucidating genotype-phenotype correlations, developing targeted therapies, and establishing international registries to improve outcomes for patients with severe *ACTA1*-related congenital myopathies.

## Data Availability

The original contributions presented in the study are included in the article/Supplementary Material, further inquiries can be directed to the corresponding author.
