# Supplementary material for: ACTA1-related congenital myopathy in a neonate: a case report and literature review
Source: Front Pediatr. 2026 Jan 20;13:1706982. doi: 10.3389/fped.2025.1706982 (PMC12864113; doi:10.3389/fped.2025.1706982)
Supplement: Supplementary file 1 [file Datasheet1.pdf]

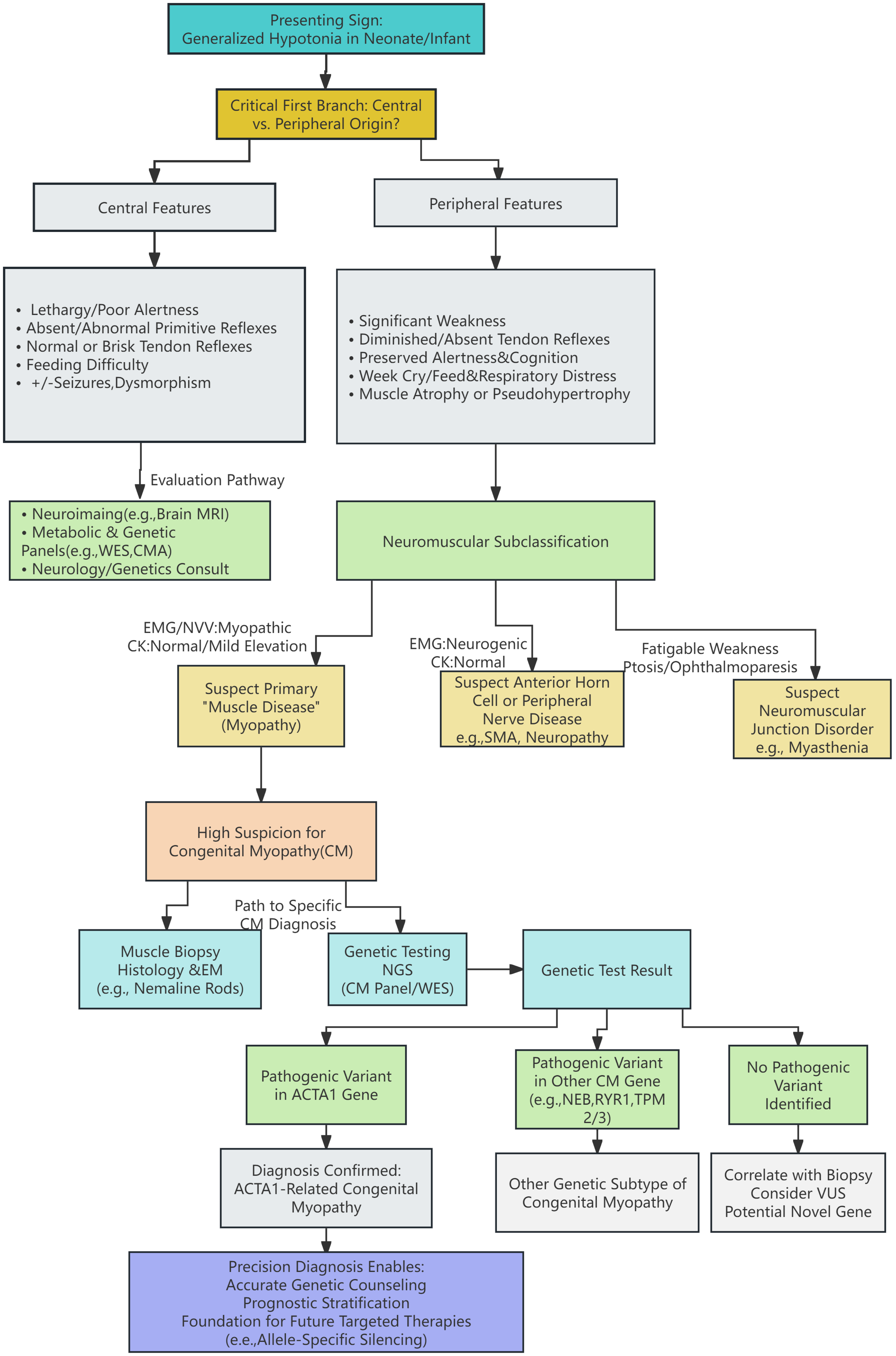
Presenting Sign:
Generalized Hypotonia in Neonate/Infant
Critical First Branch: Central vs. Peripheral Origin?
Central Features
Peripheral Features
• Lethargy/Poor Alertness
• Absent/Abnormal Primitive Reflexes
• Normal or Brisk Tendon Reflexes
• Feeding Difficulty
• +/-Seizures,Dysmorphism
• Significant Weakness
• Diminished/Absent Tendon Reflexes
• Preserved Alertness&Cognition
• Week Cry/Feed&Respiratory Distress
• Muscle Atrophy or Pseudohypertrophy
Evaluation Pathway
• Neuroimaing(e.g.,Brain MRI)
• Metabolic & Genetic Panels(e.g.,WES,CMA)
• Neurology/Genetics Consult
Neuromuscular Subclassification
EMG/NVV:Myopathic
CK:Normal/Mild Elevation
EMG:Neurogenic
CK:Normal
Fatigable Weakness
Ptosis/Ophthalmoparesis
Suspect Primary "Muscle Disease" (Myopathy)
Suspect Anterior Horn Cell or Peripheral Nerve Disease e.g.,SMA, Neuropathy
Suspect Neuromuscular Junction Disorder e.g., Myasthenia
High Suspicion for Congenital Myopathy(CM)
Path to Specific CM Diagnosis
Muscle Biopsy Histology &EM (e.g., Nemaline Rods)
Genetic Testing NGS (CM Panel/WES)
Genetic Test Result
Pathogenic Variant in ACTA1 Gene
Pathogenic Variant in Other CM Gene (e.g.,NEB,RYR1,TPM 2/3)
No Pathogenic Variant Identified
Diagnosis Confirmed: ACTA1-Related Congenital Myopathy
Other Genetic Subtype of Congenital Myopathy
Correlate with Biopsy
Consider VUS
Potential Novel Gene
Precision Diagnosis Enables:
Accurate Genetic Counseling
Prognostic Stratification
Foundation for Future Targeted Therapies
(e.e.,Allele-Specific Silencing)
